# Salinomycin induces cell death and differentiation in head and neck squamous cell carcinoma stem cells despite activation of epithelial-mesenchymal transition and Akt

**DOI:** 10.1186/1471-2407-12-556

**Published:** 2012-11-24

**Authors:** Selena Z Kuo, Katherine J Blair, Elham Rahimy, Alan Kiang, Eric Abhold, Jian-Bing Fan, Jessica Wang-Rodriguez, Xabier Altuna, Weg M Ongkeko

**Affiliations:** 1 Division of Otolaryngology-Head and Neck Surgery, Department of Surgery, University of California, San Diego, San Diego, CA, USA; 2 Illumina Inc., San Diego, CA, 92121, USA; 3 Veterans Administration Medical Center and Department of Pathology, University of California, San Diego, La Jolla, CA, USA; 4 Hospital Universitario Donostia, San Sebastian, Spain

**Keywords:** Salinomycin, Cancer stem cells, Head and neck squamous cell carcinoma, Akt, EMT, microRNA

## Abstract

**Background:**

Cancer stem cells (CSC) are believed to play a crucial role in cancer recurrence due to their resistance to conventional chemotherapy and capacity for self-renewal. Recent studies have reported that salinomycin, a livestock antibiotic, selectively targets breast cancer stem cells 100-fold more effectively than paclitaxel. In our study we sought to determine the effects of salinomycin on head and neck squamous cell carcinoma (HNSCC) stem cells.

**Methods:**

MTS and TUNEL assays were used to study cell proliferation and apoptosis as a function of salinomycin exposure in JLO-1, a putative HNSCC stem cell culture. MTS and trypan blue dye exclusion assays were performed to investigate potential drug interactions between salinomycin and cisplatin or paclitaxel. Stem cell-like phenotype was measured by mRNA expression of stem cell markers, sphere-forming capacity, and matrigel invasion assays. Immunoblotting was also used to determine expression of epithelial-mesenchymal transition (EMT) markers and Akt phosphorylation. Arrays by Illumina, Inc. were used to profile microRNA expression as a function of salinomycin dose.

**Results:**

In putative HNSCC stem cells, salinomycin was found to significantly inhibit cell viability, induce a 71.5% increase in levels of apoptosis, elevate the Bax/Bcl-2 ratio, and work synergistically with cisplatin and paclitaxel in inducing cell death. It was observed that salinomycin significantly inhibited sphere forming-capability and repressed the expression of CD44 and BMI-1 by 3.2-fold and 6.2-fold, respectively. Furthermore, salinomycin reduced invasion of HNSCC stem cells by 2.1 fold. Contrary to expectations, salinomycin induced the expression of EMT markers Snail, vimentin, and Zeb-1, decreased expression of E-cadherin, and also induced phosphorylation of Akt and its downstream targets GSK3-β and mTOR.

**Conclusions:**

These results demonstrate that in HNSCC cancer stem cells, salinomycin can cause cell death and decrease stem cell properties despite activation of both EMT and Akt.

## Background

Cancer stem cells (CSCs) are a unique subpopulation within a tumor that have the ability to self-renew and differentiate, making them responsible for initiating and maintaining tumors [1-3]. One of the main threats of CSCs is that they are resistant to conventional cancer treatments including chemotherapy and radiotherapy. Standard cancer treatments are effective in killing the bulk of the tumor but spare the CSCs, thereby progressively increasing the fraction of CSCs in the tumor [4]. The mortality of cancer remains high because conventional therapies often fail to eradicate the CSC population, allowing relapse to occur. Therefore, a complete cure for cancer likely involves treatments that can effectively eliminate CSCs along with the bulk of the tumor.

In a recent study, Gupta et al. used a high throughput screening to identify drugs that could potentially be used to target breast CSCs. By using a novel method of screening, approximately 16,000 compounds were evaluated for their ability to eradicate breast CSCs. This screening revealed that the compound salinomycin was able to kill breast CSCs 100-fold more effectively than paclitaxel [5]. Commonly, salinomycin is a monocarboxylic polyether antibiotic used to prevent coccidiosis in poultry. As an antibiotic, salinomycin functions in different biological membranes as an ionophore with a high specificity for potassium [6,7]. The antibiotic properties of salinomycin are well known, but its potential to eradicate CSCs in other cancer types needs to be further elucidated.

The epithelial-mesenchymal transition (EMT) has long been linked to the invasive properties of cancer stem cells. It is a key developmental process where immotile epithelial cells acquire mesenchymal properties and display an increased motility. It is commonly characterized by a down-regulation of E-cadherin, a critical cell-to-cell adhesion molecule [8]. An induction of EMT is directly associated with activation of the PI3K/Akt pathway, as activation of Akt has been shown to down-regulate E-cadherin in part through stabilization of the transcriptional repressor Snail [9,10]. Akt is a serine/threonine protein kinase that plays a central role in cell proliferation, growth, and survival. Akt is often found to be constitutively active in many forms of cancer, and is responsible for the anti-apoptotic properties of carcinomas [11]. Glycogen synthase kinase-3 (GSK3-β) and mTOR, two immediate downstream targets of Akt kinase activity, have previously been implicated as mediators of EMT [5,12-14].

Recent studies have shown that epithelial cells undergoing EMT acquire critical stem-cell characteristics such as the ability to self-renew [15]. Furthermore, Gupta et al. used EMT-induced breast cancer stem cells in the screening that discovered salinomycin; breast cancer cells having undergone shRNA-mediated knock-down of E-cadherin expression displayed an increased proportion of CD44^high^/CD24^low^ cells, increased resistance to chemotherapeutic drugs, and enhanced sensitivity to salinomycin [5]. Of particular significance in the context of our study, Basu et al. demonstrated that salinomycin targets mesenchymal-like cell populations within advanced-stage HNSCC. This mesenchymal subpopulation was characterized as having elevated resistance to the EGFR inhibitor cetuximab and the chemotherapeutic drugs paclitaxel and cisplatin, thus demonstrating increased drug resistance, a characteristic of cancer stem cells. The observed resistance to cisplatin in vitro and in primary-tumor derived xenografts was not present for salinomycin. [16].

The purpose of the present study was to extend our understanding of salinomycin’s therapeutic properties in head and neck squamous cell carcinoma (HNSCC) stem cells. We aim to determine whether salinomycin, alone and in combination with conventional chemotherapeutic agents, effectively induces apoptosis in HNSCC stem cells, and to further investigate its effects on cancer stem cell properties including invasion, EMT, BMI-1 expression, CD44 expression and sphere formation. CD44 and BMI-1 regulate self-renewal and have been established as CSC markers in HNSCC [17]. In addition, the effect of salinomycin on Akt signaling has not been previously examined in any cancer type. The results of this study demonstrate the ability of salinomycin to target head and neck cancer stem cells, and further examines its effects on EMT and Akt.

## Methods

### Ethics statement

Cultures used in this study (JLO-1) were derived in accordance with the policy and procedures of Hospital Donosita, San Sebastion, Spain. Tissue was obtained anonymously and all data were analyzed anonymously throughout the study, thus no patient consent was obtained. Hospital Donostia, San Sebastian approved this procurement of tissue including the waiver of consent.

### Cell lines and cell cultures

JLO-1 is a putative cancer stem cell culture derived anonymously from a fresh laryngeal tumor of patients undergoing resection of their cancer. Stem cell selective cultivation conditions for JLO-1 have been described in our previous study [18]. Briefly, flow cytometry was performed to select for CD44+ cells, which were then grown on laminin-coated plates and cultured in keratinocyte serum-free media (Invitrogen, Carlsbad, CA) containing 2 mM L-glutamine (Invitrogen), 50 μg/mL gentamycin (Invitrogen), and 20 ng/mL EGF and FGF (R&D Systems, Minneapolis, Minnesota) supplemented daily. Cultures were incubated at 37°C in 5% O_2_ and 10% CO_2_.

The established HNSCC cell lines UMSCC-10B, HN-1, and HN-30 were used in this study. UMSCC-10B was a kind gift from Dr. Tom Carey, University of Michigan, and HN-1 and HN-30 were gifts from Dr. J.S. Gutkind, National Institute for Dental and Craniofacial Research. Cell lines were routinely cultured in DMEM supplemented with 10% fetal bovine serum (FBS), 2% streptomycin sulfate (Invitrogen), and 2% L-glutamine (Invitrogen), and incubated at 37°C in 5% CO_2_ and 21% O_2_.

### Chemicals and antibodies

Salinomycin was obtained from MP Biomedicals, LLC (Solon, OH), and a 1 mM stock solution was prepared in 100% ethanol. Prior to cell treatment, working concentrations of salinomycin were prepared in culture media. Control groups were treated with an equal volume of ethanol vehicle. Cisplatin and paclitaxel were purchased from Sigma-Aldrich (St. Louis, MO). Rabbit polyclonal Bax, Rabbit polyclonal Bcl-2, Rabbit polyclonal p-Akt (Ser473), rabbit monoclonal vimentin (D21H3) XP, rabbit monoclonal p-GSK3β (Ser9), rabbit polyclonal p-mTOR (Ser2448), and rabbit polyclonal total ERK antibodies were from Cell Signaling (Beverly, MA). Rabbit polyclonal Snail antibody was obtained from Abcam (Cambridge, MA).

### Flow cytometry

Flow cytometry was used to confirm the CD44+ population of the putative head and neck cancer stem cell population. Cells were trypsinized and incubated with anti-human CD44-APC antibody (BD Biosciences) or a non-specific IgG antibody as a negative control.

### Cell proliferation assay

MTS assays were performed using the CellTiter 96 Aqueous non-radioactive cell proliferation assay (Promega, Madison, WI). Cells were trypsinized, counted, and replated into a 96-well plate at 5000 cells per well. Cells were allowed to adhere overnight. To generate a dose–response curve for salinomycin, indicated doses of salinomycin were added to the corresponding wells for an incubation period of 48 hours. For synergistic assays involving the combination of cisplatin and salinomycin, cells were treated with 4 μM of salinomycin for 48 hours followed by co-treatment with cisplatin at a range of doses (1, 2, 5, 10, 20 μM) for an additional 48 hours. For synergistic assays involving the combination of paclitaxel and salinomycin, cells were treated with 0.5 μM of salinomycin for 48 hours followed by co-treatment with paclitaxel at a range of doses (1, 2, 3, 4, 6, 8 nM) for an additional 48 hours. Each permutation was performed in triplicates. Following the indicated incubation periods for the above assays, 20 μL of the MTS reagent was added into each well followed by a 1–3 hour incubation period. The plates were then read at an absorbance of 490 nm.

### Combination index analysis of drug interactions

To determine whether the observed cytotoxic interactions of salinomycin with paclitaxel/cisplatin were synergistic, additive, or antagonistic in nature, the combination index (CI) method of Chou and Talalay was used [19]. The CI value is a quantitative measure indicating the type of interaction between two drugs: CI <1 indicates synergism, CI = 1 indicates an additive effect, and CI > 1 indicates antagonism. The CI value for each experimental group was calculated using the following formula: CI = (D)_1_/(D)_2_ + (D_x_)_1_/(D_x_)_2_, where (D)_1_ and (D)_2_ in the numerator are the concentrations of drug 1 and 2 required in combination to produce a survival of x%, and (D_x_)_1_ and (D_x_)_2_ in the denominator are the concentrations of drug 1 and 2 required to individually produce a survival of x%.

### Trypan blue dye exclusion assay

In order to confirm the observed synergy between salinomycin and cisplatin/paclitaxel, a trypan blue exclusion assay was performed for the combination treatment which generated the lowest CI value (indicative of the greatest synergy) and produced a survival of less than 80%. Cells were pre-treated with indicated doses of salinomycin (4 μM for cisplatin + salinomycin combination treatments and 0.5 μM for paclitaxel + salinomycin combination treatments) followed by co-treatment with paclitaxel (3 nM) or cisplatin (5 μM) for an additional 48 hours. Media was replenished following initial salinomycin pre-treatment. Cell viability for each experimental group was then determined by the percentage of cells that excluded the dye, as trypan blue only traverses the membrane of dead cells. Cells were mixed with an equal volume of 0.4% trypan blue dye, and allowed to incubate for 5 minutes. The percentage of trypan blue positive cells was then determined by manually counting the stained fraction with a hemocytometer.

### TUNEL assay

Cells were treated with salinomycin 4 days prior to fixing in 70% Ethanol. Media and growth factors were not replenished throughout the treatment. Using the APO-BRDU^TM^Kit (Phoenix Flow Systems, Inc., San Diego, CA), the cells undergoing apoptosis were labeled with bromolated deoxyuridine triphosphate nucleotides (BrdUTP). These cells were then identified and binded to a fluorescein labeled antiBrdU monoclonal antibody. After the required incubation times, the samples analyzed for the proportion of apoptotic cells by flow cytometry.

### Quantitative real-time PCR

The cultured cells were treated with salinomycin (0 – 8 μM) for 48 hours. Total cell lysate was collected and mRNA was extracted using the RNeasy kit (QIAGEN). cDNA was then synthesized from 1.5 μg of total mRNA using reverse transcriptase (Invitrogen, Carlsbad, CA, USA), as per the manufacturer’s instructions. Real-time quantitative PCR was performed by combining 2.5 μl of the RT with 22.5 μl of SYBR green (Roche, Basel, Switzerland). The reaction was run using System 7300 (Applied Biosystems, Foster City, CA, USA) and results were analyzed by the relative quantity method. Experiments were performed in triplicates with GAPDH expression as the endogenous control. Primers were custom designed by the authors and created by Operon Biotechnologies, Alabama, USA. The following sequences were used: GAPDH forward: 5^′^-CTTCGCTCTCTGCTCCTCC-3^′^ GAPDH reverse: 5^′^-CAATACGACCAAATCCGTTG-3^′^ CD44 forward: 5^′^-ACACCACGGGCTTTTGACCAC-3^′^ CD44 reverse: 5^′^-AGGAGTTGCCTGGATTGTTGCTTG-3^′^ BMI-1 forward: 5^′^-TCCACAAAGCACACACATCA-3^′^ BMI-1 reverse: 5^′^-CTTTCATTGTCTTTTCCGCC-3^′^ Snail forward: 5^′^-CTGCCCTGCGTCTGCGGAAC-3^′^ Snail reverse: 5^′^-GCTTCTCGCCAGTGTGGGTCC-3^′^ E-Cadherin forward: 5^′^-CTGATGTGAATGACAACGCC-3^′^ E-Cadherin reverse: 5^′^-TAGATTCTTGGGTTGGGTCG-3^′^ ZEB-1 forward: 5^′^-GCCGCTGTTGCTGATGTGGCT-3^′^ ZEB-1 reverse: 5^′^-TCTTGCCCTTCCTTTCCTGTGTCA-3^′^ ALDH1A1 forward: 5^′^-CGCCAGACTTACCTGTCCTA-3^′^ ALDH1A1 reverse 5^′^-GTCAACATCCTCCTTATCTCCT-3^′^ Oct-4 forward: 5^′^-GCAAAGCAGAAACCCTCGTGC-3^′^ Oct-4 reverse: 5^′^-ACCACACTCGGACCACATCCT-3^′^ Nanog forward: 5^′^-GATTTGTGGGCCTGAAGAAA-3^′^ Nanog reverse: 5^′^-TTGGGACTGGTGGAAGAATC-3^′^.

### Tumor sphere formation assay

The putative cancer stem cell cultures were plated at a density of 500 cells/ml in a low-adhesion tissue culture plate. Serum free media containing 25 ng/ml growth factors (1/5^th^ normal growth factor concentration) was used. Salinomycin was added when the cells were plated at concentrations of 0, 0.5, 1, 2, 4, 8 μM. Salinomycin was re-added every other day for 10 days and on day 10 the spheres were photographed. Media and growth factors were not replenished throughout the assay. Spheres were plated and counted in quadruplicates.

### Invasion assay

Inserts with 8 μm pores (BD Biosciences) were coated with Matrigel from EHS murine sarcoma (Sigma), at a concentration of 3 mg/mL. Cells were pretreated with their respective concentrations of salinomycin for 4 days and 100,000 viable cells of each permutation were added to their respective inserts. To ensure that perceived changes in invasion were not due to cytotoxicity of salinomycin, an MTS was performed for JLO-1 cells under the same conditions as the Salinomycin-treated cells. Cell numbers were then adjusted according to the MTS data to account for discrepancies in cell death by using the following formula: (100,000)/(x) = (% cell viability)/(100), where (x) = number of cells added into each insert and (% cell viability) is determined by the MTS (i.e., treatment with 4 μM resulted in% cell viability of 33.0%; thus 303,030 cells were added into their respective inserts.). Each permutation was performed in triplicates. Cells were left to invade for 48 hours under hypoxic conditions (5% O_2_). After 48 hours, cells were fixed for 2 minutes in 100% methanol and then stained in crystal violet. Cells that invaded were counted in a pre-determined field.

### Western blot analysis

Respective doses of salinomycin were added to the cells 48 hours before harvesting. Cells were lysed on ice for 10 minutes with RIPA buffer (0.1 M Tris, 2% SDS, 20% glycerin, and protease inhibitor tablets from Roche Diagnostics, Indianapolis, IN). Gel electrophoresis using 10% NuPage Bis-Tris gels separated the proteins, which were then transferred onto a PVDF membrane. The membrane was blocked for one hour in 5% non-fat dry milk in TBST and incubated overnight in primary antibody at a dilution of 1:1,000. The membranes were then incubated in their appropriate secondary antibodies at a dilution of 1:10,000 and each specific protein was visualized using SuperSignal West Pico Luminol (Pierce, Rockford, IL).

### MicroRNA profiling

MicroRNA was isolated using the mirVana miRNA isolation kit (Ambion, Austin, TX), following the manufacturer’s instructions. Samples were run on the Illumina MicroRNA Array Profiling platform [20]. Analyses were performed using BRB-ArrayTools developed by Dr. Richard Simon and BRB-ArrayTools development team. Clustering algorithms were performed by Cluster 3.0 and visualized with TreeView (Eisen Lab, Stanford University). The data discussed in this study have been deposited in NCBI’s Gene Expression Omnibus [21] and are accessible through GEO Series accession number GSE33196 (http://www.ncbi.nlm.nih.gov/geo/query/acc.cgi?acc=GSE33196). Candidate microRNAs were identified and confirmed by RT-qPCR with microRNA-specific forward primers and a universal reverse primer. U6 small nuclear RNA transcript served as the normalization signal. The sequences of RT-qPCR primers for microRNA detection were as follows: hsa-mir-328: 5^′^-CTGGCCCTCTCTGCCCTTCCGT-3^′^ hsa-mir-203: 5^′^-GTGAAATGTTTAGGACCACTAG-3^′^ hsa-mir-199a-3p: 5^′^-ACAGTAGTCTGCACATTGGTTA-3^′^ Universal reverse: 5^′^-GCGAGCACAGAATTAATACGACT-3^′^ U6 forward: 5^′^-GGGGACATCCGATAAAATTGG-3^′^ U6 reverse: 5^′^-ACCATTTCTCGATTTGTGCGT-3^′^.

### Data analysis

Results represent mean and SD where appropriate. Experiments were performed in duplicate (western blot and TUNEL) or triplicate.

## Results

### Acquisition of a cancer stem cell culture

**Figure 1:**
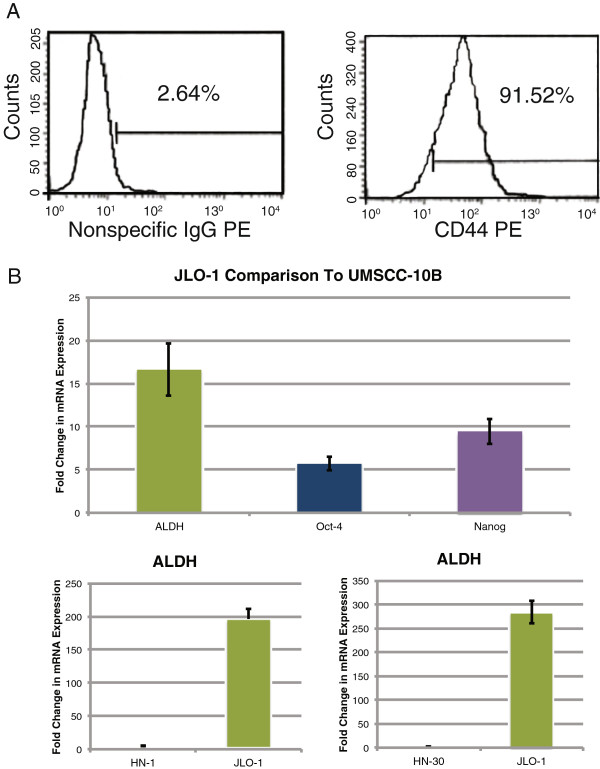
**Isolation of HNSCC stem cell culture. **(**A**) Flow cytometry confirms that our isolated cell culture is 91.5% CD44 positive. A nonspecific IgG antibody was used as a negative control. (**B**) RT-qPCR further confirms the stem cell characteristics of JLO-1 by showing elevated ALDH levels in comparison to three control cell lines (UMSCC-10B, HN1, and HN30). JLO-1 also has increased levels of Oct-4 and Nanog relative to UMSCC-10B.

A putative cancer stem cell culture, JLO-1, was derived from a fresh laryngeal cancer tissue. Cells were cultured for several months under conditions that favored the growth of stem cells and inhibited the growth of bulk tumor cells. The culture was confirmed to be 91.5% CD44 positive by flow cytometry (Fig. 1A). To further verify the stem cell phenotype of the JLO-1 culture, a qPCR was performed to evaluate the expression of aldehyde dehydrogenase class-1A1 (ALDH1A1) and the transcription factors Oct-4 and Nanog in JLO-1 relative to a HNSCC cell line, UMSCC-10B, cultured under standard conditions. Previous studies indicate ALDH is a more specific HNSCC CSC marker than CD44, as ALDH expression identifies a subpopulation of CD44 positive cells containing the tumorigenic cancer stem cells [22,23]. JLO-1 demonstrated considerably higher expression of ALDH, Oct-4, and Nanog relative to UMSCC-10B (Fig. 1B). ALDH1A1 expression of JLO-1 relative to two additional HNSCC cell lines was assessed for further verification (Fig. 1B).

### Salinomycin induces a dose-dependent increase in cell death

**Figure 2:**
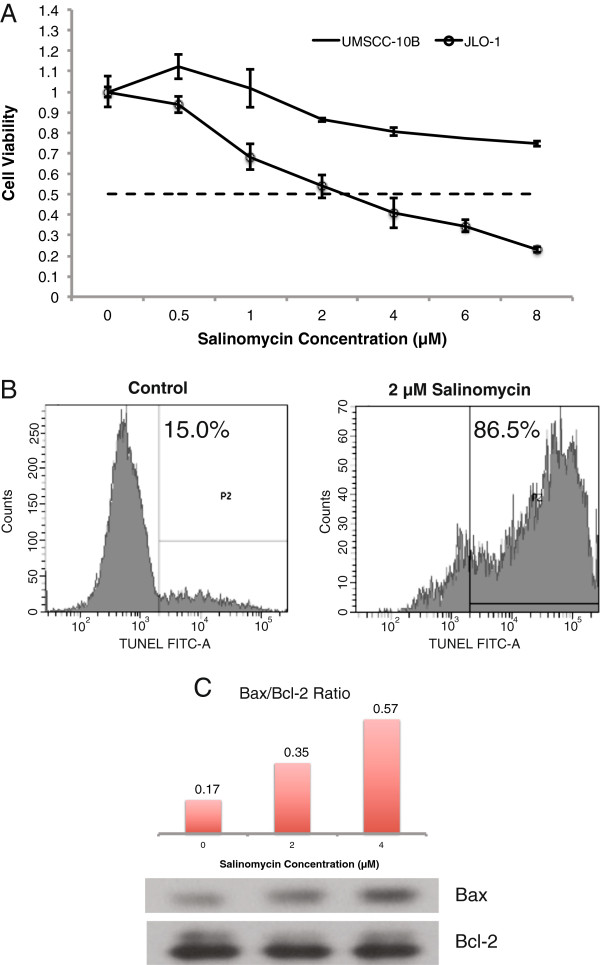
**Salinomycin causes a decrease in cell viability and induces apoptosis. **(**A**) MTS assay shows salinomycin causes a selective decrease in cell proliferation of JLO-1 compared to UMSCC-10B. The absorbance values (Y-axis) were normalized by dividing over the absorbance of each control. Error bars represent standard deviation. (**B**) TUNEL assay shows an increase in apoptosis with a 2 μM salinomycin treatment indicated by the percent increase in DNA strand breaks. (**C**) Western blot demonstrates a dose dependent increase in apoptosis as seen by the induction in Bax/Bcl-2 ratio.

To determine the effects of salinomycin on the HNSCC stem cells, an MTS assay was performed to measure changes in cell proliferation and viability. A range of doses (0 – 8 μM) previously published by Gupta et al. was used to quantify cell death after 48 hours. JLO-1 experienced significant toxicity towards salinomycin in a dose dependent manner, with an IC_50_ close to 2 μM. In a parallel experiment, UMSCC-10B exhibited less sensitivity to salinomycin treatment, with an IC_50_ beyond 8 μM (Fig. 2A). To further verify cell death, a TUNEL assay was performed to measure amounts of DNA strand breaks, which correspond to the levels of apoptosis caused by salinomycin. At 2 μM, there was a substantial increase in the proportion of CSCs undergoing apoptosis compared to the control (Fig. 2B). Western blot analysis revealed increasing protein levels of pro-apoptotic bax and constant levels of anti-apoptotic bcl-2 upon salinomycin treatment, indicating a dose-dependent increase in the Bax/Bcl-2 ratio and greater mitochondrial permeabilization (Fig. 2C). Our results are consistent with those of Basu et al. suggesting salinomycin effectively kills treatment-resistant malignant subpopulations in HNSCC [16].

### Salinomycin synergistically increases cell death in combination with cisplatin and paclitaxel

**Figure 3:**
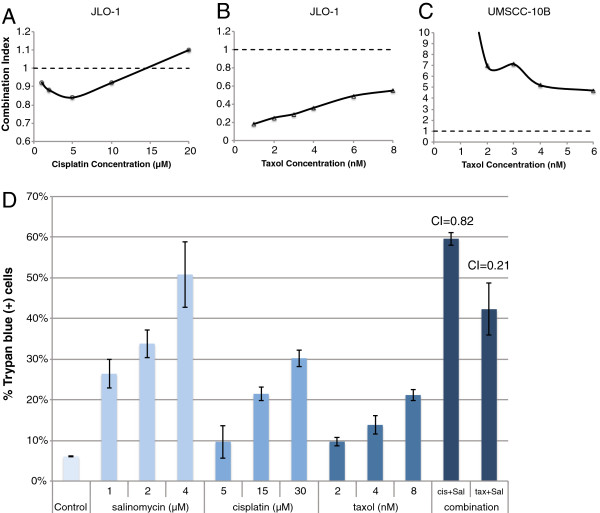
**Combination treatments with salinomycin and chemotherapy drugs synergistically target cancer stem cells. **The mean combination index (CI) value of combination treatments in JLO-1 were calculated as explained in the *Methods*. CI < 1 indicates synergy, CI = 1 (denoted by dashed line) indicates additivity, and CI > 1 indicates antagonism. (**A**) CI graph depicts cytotoxic interactions between 4 μM salinomycin and increasing doses of cisplatin (1, 2, 5, 10, 20 μM) in JLO-1. (**B**) CI graph depicts cytotoxic interactions between 0.5 μM salinomycin and increasing doses of taxol (1, 2, 3, 4, 6, 8 nM) in JLO-1. (**C**) CI graph depicts cytotoxic interactions between 0.5 μM salinomycin and increasing doses of taxol (1, 2, 3, 4, 6, 8 nM) in a parallel experiment for UMSCC-10B. (**D**) Trypan blue dye exclusion assay further verifies observed synergy for JLO-1 receiving combination treatments of 0.5 μM salinomycin + 3nM taxol or 4 μM salinomycin + 5 μM cisplatin. Calculated CI values are shown above respective bars. All error bars represent standard deviation.

Since salinomycin shows promise as a novel treatment for cancer, we sought to determine which chemotherapy drugs would be beneficial for concurrent treatment. We tested the synergy between salinomycin and two conventional chemotherapy drugs for HNSCC: cisplatin and paclitaxel. MTS assays were performed to compare the differences in the survival curves between each chemotherapy drug alone and the combination treatments. Using the Chou-Talalay combination index (CI) method, we observed synergistic cytotoxic interactions between salinomycin and both chemotherapeutic drugs (Fig. 3A and B). However, paclitaxel exhibited stronger synergism with salinomycin, as indicated by lower CI values. Interestingly, in a parallel experiment with UMSCC-10B, paclitaxel and salinomycin exhibited an antagonistic drug interaction (Fig. 3C). To further confirm the observed cytotoxic synergism in JLO-1, a trypan blue exclusion dye assay was performed for the combination treatment exhibiting the lowest CI value (greatest synergism) that induced cytotoxicity of at least 20%. Combination treatment of 5 μM cisplatin and 4 μM salinomycin resulted in a CI of 0.82, while combination treatment of 3 nM paclitaxel and 0.5 μM salinomycin resulted in a CI of 0.21 (Fig. 3D). As the CI values are below 1 (1 indicates additivity), the results demonstrate that both combination treatments synergistically targeted the CSC population more efficiently than either drug alone, although paclitaxel exhibits markedly greater synergism than cisplatin.

### Salinomycin decreases stem cell markers and self-renewal capabilities

**Figure 4:**
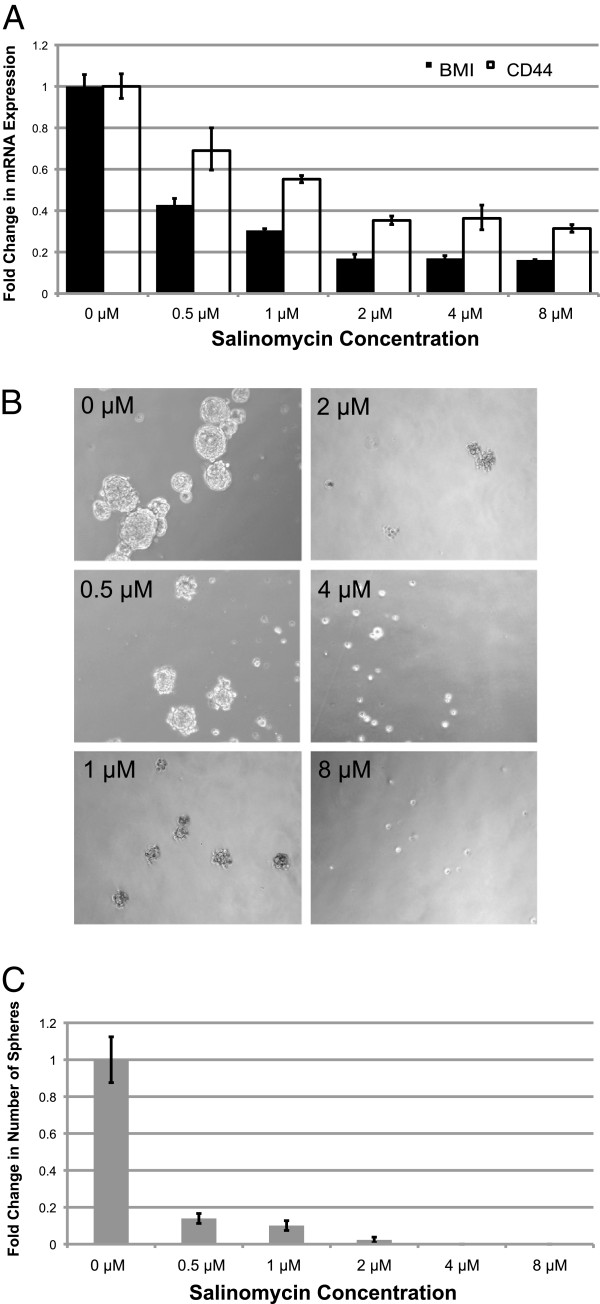
**Salinomycin decreases expression of stem cell markers and self-renewal properties. **(**A**) The RT-qPCR results demonstrate a decrease in gene expression of both CD44 and BMI-1 with increasing doses of salinomycin. Values are relative to a control of 0 μM salinomycin and endogenous control GAPDH. (**B**) Sphere formation assay shows that salinomycin inhibits self-renewal capabilities of the cancer stem cells. Salinomycin was added during sphere growth. (**C**) Accompanying graph shows the fold change in number of spheres formed relative to the control of 0 μM salinomycin. Error bars denote standard deviation.

To determine if salinomycin also causes a decrease in stem cell capabilities, a RT-qPCR was performed to quantify the change in gene expressions of the known markers BMI-1 and CD44 were measured. CD44 is a well-documented cell surface marker for head and neck cancer and BMI-1 is necessary for self-renewal. Using the same range of doses, the results showed a dose-dependent decrease of CD44 and BMI-1, both of which are critical for maintaining tumorigenicity in head and neck CSCs (Fig. 4A). To confirm these effects, a sphere formation assay was performed. The ability to form spheres is a defining feature and indicator of CSCs. Salinomycin was added during sphere formation, and the substantial decrease in number of spheres formed confirms that salinomycin inhibits self-renewal of CSCs. At the highest doses (4 μM and 8 μM) no spheres were formed (Fig. 4B and C).

### Salinomycin induces EMT but decreases invasive abilities

**Figure 5:**
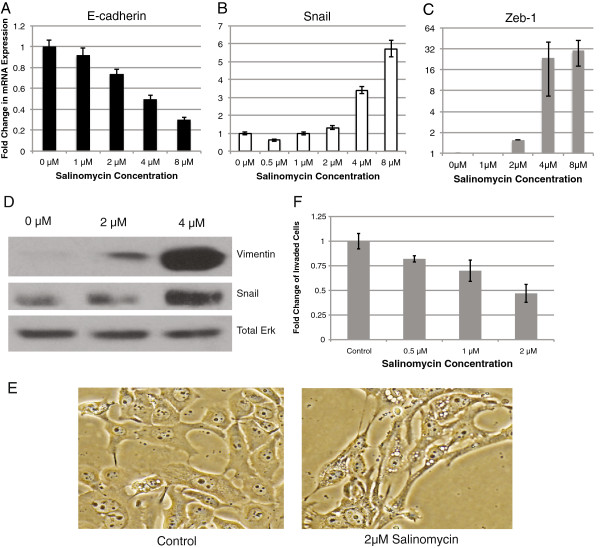
**Salinomycin induces EMT but decreases invasive properties. **(**A**-**C**) The RT-qPCR data shows a decrease gene expression in E-cadherin and an upregulation of Snail and Zeb-1 as labeled, which correspond to an induction of EMT. All data is relative to the control of 0 μM salinomycin and endogenous control GAPDH. (**D**) Western blotting confirms the induction of Snail and shows an upregulation of the mesenchymal marker vimentin. (**E**) Micrographs of JLO-1 upon treatment with 2 μM salinomycin depicts alterations in cell morphology. (**F**) The graph denotes the fold change in number of cells that invaded through a matrigel membrane relative to the control of 0 μM salinomycin. Error bars represent standard deviation.

The ability to invade and metastasize is a characteristic of CSCs that is often enabled by EMT. Recent studies have even shown a direct link between an induction of EMT and a gain in stem cell properties such as self-renewal. Therefore, we sought to determine the effects of salinomycin on EMT by examining the changes in the known regulatory markers E-cadherin, Zeb-1, Snail, and vimentin. Contrary to our hypothesis, salinomycin caused an induction of EMT. As shown by RT-qPCR, there is a substantial increase in expression of Snail and Zeb-1 and decrease in epithelial marker E-cadherin (Fig. 5A-C). Immunoblotting verified the increase in Snail and further established the induction of EMT by indicating an increase in the mesenchymal marker vimentin. (Fig. 5D). In addition, treatment with 2 μM salinomycin resulted in the acquisition of a spindle-shaped cell morphology (Fig. 5E). As induction of EMT was accompanied by increasing amounts of cell death, we speculated whether the observed EMT was simply an epiphenomenon triggered by significant cell death as opposed to a salinomycin-specific response. To exclude this possibility, JLO-1 was treated with cytotoxic levels of a control drug (one that does not influence EMT at non-cytotoxic doses), and changes in EMT genes were assessed. Cell death was shown to have marginal to no effect on EMT in JLO-1 cells (Additional File 1). Given the surprising activation of EMT, an invasion assay was then performed to further assess the effect of salinomycin on migration. Interestingly, in disconnect with the induction of EMT, salinomycin caused a dose-dependent decrease in number of cells migrating through a matrigel membrane (Fig. 5F).

### Salinomycin induces phosphorylation of Akt

**Figure 6:**
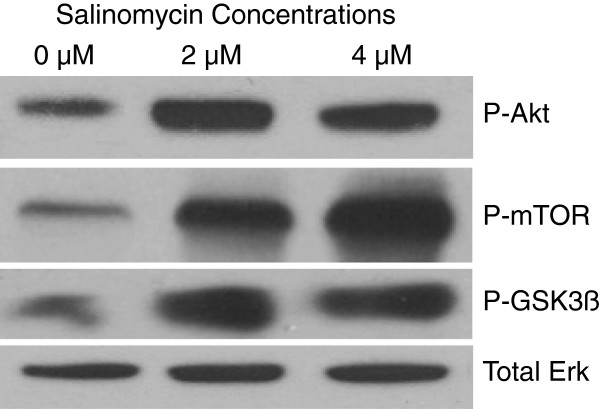
**Salinomycin induces phosphorylation of Akt. **Western blotting shows an increase in phosphorylation of Akt (Ser473), as well as the immediate downstream targets GSK3-β (Ser9) and mTOR (Ser2448), when treated with the indicated doses of salinomycin. Total Erk is utilized as a loading control.

The activation of the PI3K/Akt pathway has been shown to be a central feature of EMT. This signaling pathway is often found overly active in many cancers, which negatively influences prognosis. In search of an explanation and further verification of the unanticipated increase in EMT markers, we investigated the effects of salinomycin on Akt. Consistent with our EMT results, salinomycin caused an increase in phosphorylation of Akt (Fig. 6). Activated Akt has been shown to result in the inhibition of Bax and up-regulation of Bcl-2, in contrast to Figure 2C. Thus, to verify that phosphorylation of Akt in fact correlated with increased kinase activity, we investigated the phosphorylation status of two immediate downstream effectors implicated in EMT, GSK3-β and mTOR. Previous studies have identified Snail as a direct target of active (unphosphorylated Ser-9) GSK3-β, resulting in inhibition of snail transcription and promotion of snail degradation [12,13]. Immunoblotting revealed increased phosphorylation of GSK3-β and mTOR. Taken together, our findings indicate that the induction of EMT follows an increase in activation of Akt, but the levels of cell death caused by salinomycin are independent of this anti-apoptotic pathway.

### Salinomycin induces changes in microRNA Expression

**Figure 7:**
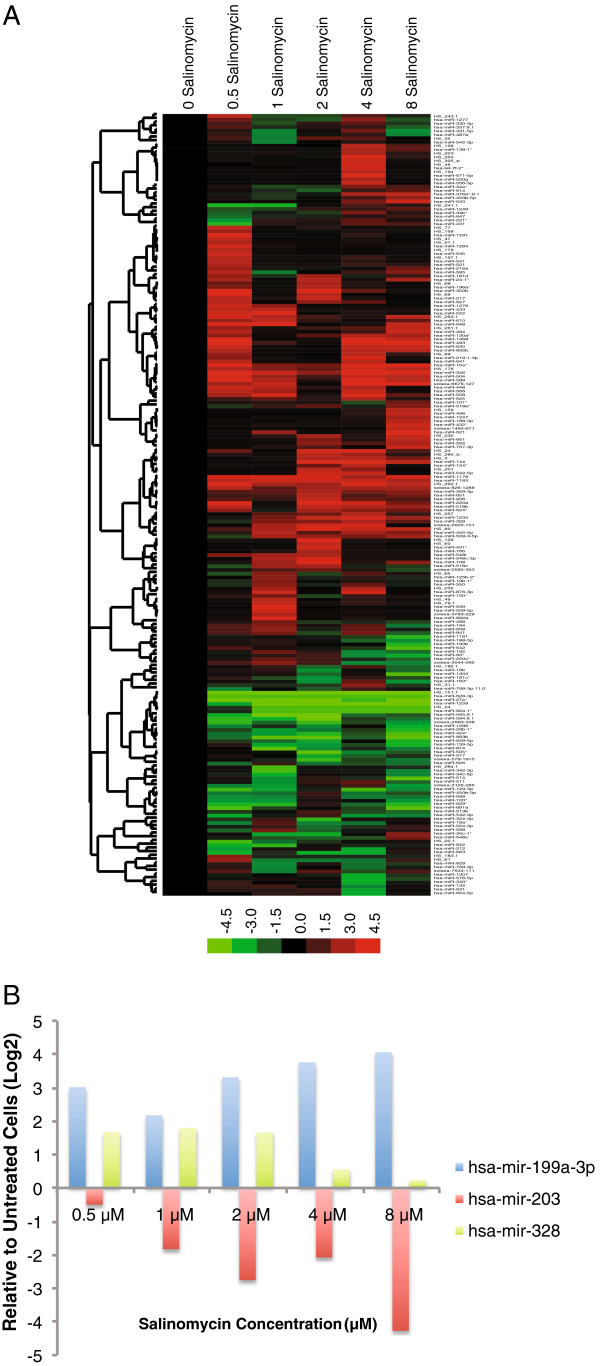
**Salinomycin induces changes in microRNA expression. **(**A**) Heatmap derived from microarray data showing sets of microRNAs up- or down-regulated by salinomycin. (**B**) Three candidate microRNAs identified by microarray were further verified via RT-qPCR. Data is shown as the mean results of two separate experiments.

MicroRNAs have gained widespread attention for their roles in regulating many aspects of cancer progression including EMT, invasion and stem cell properties. To determine whether the effect of salinomycin could potentially be mediated by microRNA activity, we performed a microarray analysis of global microRNA expression in JLO-1 cells treated with increasing doses of salinomycin. Clustering analysis revealed a set of microRNAs that were consistently up or down-regulated by salinomycin, suggesting that the effects of salinomycin may potentially be mediated through changes in microRNA expression (Figure 7a). Among these microRNAs were miR-328 and miR-199a-3p (Figure 7b), both with known roles in promoting drug sensitivity [24-26]. Interestingly, salinomycin downregulated the expression of miR-203, which is known to inhibit EMT [27].

## Discussion

The CSC-inhibiting activity of salinomycin has previously been demonstrated in a variety of tumors including those of the breast, lung, and colon. Here we have extended these studies by showing that salinomycin induces apoptosis and chemosensitivity while inhibiting cell proliferation, invasion, stem cell marker expression and sphere formation in putative HNSCC stem cells. Ultimately, these results suggest that salinomycin or its derivatives may be an effective novel treatment for HNSCC, especially when administered in combination with standard treatments. Our results are consistent with a previous study by Busa et al. reporting the ability of salinomycin to eradicate treatment-resistant phenotypes in HNSCC. However, Basu et al. report no observed synergistic efficacy between salinomycin and cisplatin in HNSCC in vitro, speculating a possible overlap of the individual drugs’ cytotoxic mechanisms [16]. Although the method of quantifying drug interactions is not specified, we are not surprised by this finding given the relatively weak synergy observed between cisplatin and salinomycin in JLO-1. In contrast, combination treatment of paclitaxel with salinomycin resulted in strong synergy for all tested drug ratios, emphasizing the potential of this drug pair in the treatment of HNSCC. Salinomycin was also observed in our system to activate Akt signaling and induce changes in gene expression indicative of EMT. These results are quite unusual and potentially worrisome given that Akt signaling and EMT are both heavily implicated in cell proliferation, invasion and acquisition of CSC properties.

At this time of writing there appears to be no other study which documents the effect of salinomycin on Akt, leaving open for investigation whether salinomycin also activates Akt in other cancers. Drugs including cisplatin, etoposide, doxorubicin, and tamoxifen have been shown to induce Akt phosphorylation leading to chemoresistance in some cancers [28-30]. Similarly, it is possible that pro-survival mechanisms within HNSCC stem cells activate Akt in the presence of salinomycin in attempt to overcome drug-induced cell death. Further investigation is required to elucidate the mechanisms that are responsible for drug-induced phosphorylation. What is clear, however, is that salinomycin is ultimately capable of inducing apoptosis and inhibiting cell proliferation in HNSCC stem cells. Since apoptosis occurs despite the activation of Akt, it is likely that salinomycin targets apoptotic pathways that are downstream of Akt. We report an induction of Bax and constant expression of Bcl-2 in salinomycin-treated JLO-1 despite increased Akt kinase activity. Previous studies have also shown that salinomycin is capable of inducing apoptosis through a variety of targets including Bcl-2, P-glycoprotein, 26S proteasome, calpain and cytochrome C, all of which are downstream or independent of Akt [31].

EMT has been nearly synonymous with the acquisition of an invasive and metastatic phenotype and its link to cancer stem cell properties is also becoming well-established [15,32]. Furthermore, salinomycin was originally identified as a cancer stem cell inhibitor by screening for drugs with specific toxicity against mesenchymally transdifferentiated breast cancer cells [5]. Likewise, Basu et al. demonstrated in vivo depletion by salinomycin of the vimentin-positive subpopulation and enrichment of the E-cadherin-positive subpopulation in primary tumor-derived xenografts, possibly through selective cytotoxicity, promotion of MET, or inhibition of EMT [16]. Thus, it is interesting that salinomycin induces gene expression changes indicative of EMT in HNSCC stem cells, especially while inhibiting invasion and stemness. This surprising observation requires a reassessment of the link between EMT and cancer stem cells, and strongly suggests that EMT may not in all cases lead to an invasive or stem cell-like phenotype.

Although this study marks the first instance in which salinomycin is shown to induce EMT, it is not the first to show a disconnection between EMT and stem cells. In fact, it is well known that embryonic stem cells (ESCs) resemble epithelial cells and have high expression of E-cadherin, which is crucial for pluripotency in ESCs and may even be used in place of Oct-4 during somatic cell reprogramming [33]. It is also well established that the reverse of EMT, mesenchymal-epithelial transition (MET), is a critical step for reprogramming mouse fibroblasts to induced pluripotent stem cells (iPSCs) [34,35]. In terms of cancer, it was recently demonstrated that prostate cancer stem cells are characterized by high E-cadherin expression, are highly invasive, and exhibit high expression of stem cell markers Oct-3/4 and Sox2 compared to cells with low E-cadherin expression [36,37]. It has been hypothesized that cancer stem cells possess a high degree of plasticity, and that following EMT, E-cadherin expression may be restored without losing stem cell function or invasive capacity [36].

Further research is required to reconcile the apparent inconsistency between contexts in terms of the relationship between EMT and stemness. Contrary to the data presented here, a previous report has confirmed the ability of salinomycin to reverse EMT in colorectal cancer [38]. Thus, whether salinomycin promotes or inhibits EMT varies between cases and may be highly dependent on cell type. In any case, the data presented here make it clear that EMT does not always correspond to stem cell phenotype or invasion, and that salinomycin may induce loss of stemness through pathways that are independent of EMT.

Investigating changes in microRNA expression may offer additional insight into the mechanism of salinomycin. In particular, microRNAs have previously been shown to regulate invasion via EMT-independent pathways [39]. MiR-328 has been shown to negatively regulate the expression of ABCG2 in human cancer cells, while miR-199a-3p has been shown to induce cell cycle arrest, reduce invasion, and increase doxorubicin sensitivity by negatively regulating mTOR and c-Met [25,26]. Interestingly, we report an increase in activation of mTOR upon salinomycin treatment despite induction of miR-199a-3p. However, it is known that individual microRNAs can target multiple components within one signaling pathway. MiR-199a-3p has also been shown to inhibit proliferation by negatively regulating the cancer stem cell marker CD44 [24]. The upregulation of these miRs may explain some of the effects of salinomycin including the downregulation of CD44, decrease in invasion, and the synergy observed between salinomycin and cisplatin or paclitaxel. The ability of salinomycin to induce EMT in HNSCC stem cells may be explained by the dose-dependent downregulation of miR-203, which has been shown to inhibit EMT in prostate cancer [27]. Further characterization of these microRNAs and other potential pathways affected by salinomycin will provide a greater understanding of how to target cancer stem cells.

## Conclusions

The results of this study lend promise to the notion of targeting cancer stem cells with small molecules. Consistent with a prior study in breast cancer, we have shown that salinomycin induces apoptosis and chemosensitivity while inhibiting cell proliferation, invasion, stem cell marker expression and sphere formation in putative HNSCC stem cells. Microarray analysis revealed that increased chemosensitivity could potentially be mediated through changes in certain microRNA levels. Contrary to the above effects and to current understanding of cancer stem cell biology, salinomycin also activated Akt signaling and induced changes in gene expression indicative of an EMT. This can be worrisome if the purpose of this drug is to inhibit proliferation and invasion/metastasis. Thus, a more complete understanding of the biological effects of salinomycin is a prerequisite to translating this compound or potential derivatives for use in a clinical setting. In addition, there is a potential need to re-investigate the relationship between stem cell phenotype, EMT and Akt signaling.

## Abbreviations

CSC: Cancer stem cell; HNSCC: Head and neck squamous cell carcinoma; EMT: Epithelial to mesenchymal transition; miRNA: microRNA.

## Competing interests

The authors declare that they have no competing interests.

## Authors’ contributions

SZK performed the qPCR for EMT genes, western blots, MTS assays, TUNEL assay, prepared the figures and wrote the manuscript excluding the discussion. KJB performed the qPCR for stem cell genes, sphere formation assay, matrigel invasion assay, and drafted the first version of the manuscript. ER performed additional qPCR assays, western blots, trypan blue assays, MTS assays, assisted in data analysis, and revised the manuscript. AK prepared the microRNA for analysis, wrote the discussion, and revised the manuscript. EA assisted SZK in many of the experimental assays and helped analyze data. JBF performed the microarray for miRNA expression. JWR participated in design and coordination of this study. XA derived the putative CSC and helped analyze the data. WMO conceived of the study, supervised the entire project, analyzed the data, and revised the manuscript. All authors read and approved the final manuscript.

## Pre-publication history

The pre-publication history for this paper can be accessed here:

http://www.biomedcentral.com/1471-2407/12/556/prepub

## Supplementary Material

Additional File 1 **Format: PDF. **Cell death does not significantly alter expression of EMT and stem cell genes in JLO-1. A drug control was used to confirm that dose-dependent induction of EMT genes and repression of stem cell genes was not a mere epiphenomenon of cell death accompanying salinomycin treatment. We have previously discovered that Metformin does not influence EMT in JLO-1 cells at non-cytotoxic doses, indicating it does not regulate EMT in JLO-1 (unpublished data). (A) An MTS assay was initially performed to determine the cytoxicity curve for JLO-1 cells treated with Metformin for 72 hours. (B) At non–cytotoxic concentrations, Metformin does not regulate EMT based on RT-qPCR data of Snail and E-cadherin transcript levels, thus it is an appropriate drug control to induce cell death in JLO-1. (C) Upon 48-hour treatment of JLO-1 with 15 mM Metformin to induce approximately 60% cell death (equivalent to the cell death observed from 4 μM salinomycin treatment), expression of EMT genes Snail and E-cadherin showed only minor changes. In addition, CD44 expression was not effected by induction of cell death. Click here for file
