# Supplementary figures and images for: Salinomycin induces cell death and differentiation in head and neck squamous cell carcinoma stem cells despite activation of epithelial-mesenchymal transition and Akt
Source: BMC Cancer. 2012 Nov 24;12:556. doi: 10.1186/1471-2407-12-556 (PMC3522015; doi:10.1186/1471-2407-12-556)

# A Metformin Treatment of JLO-1

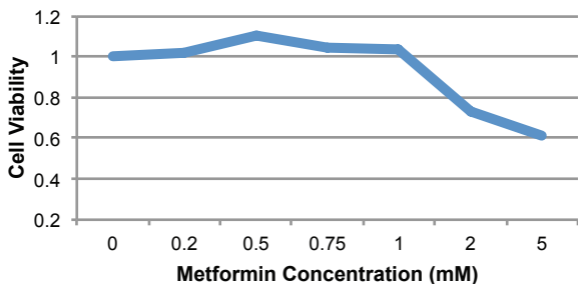

B

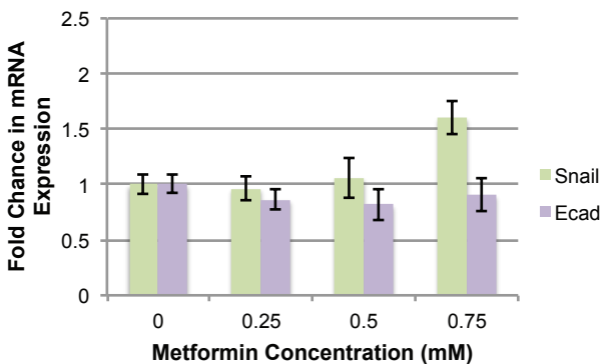

C

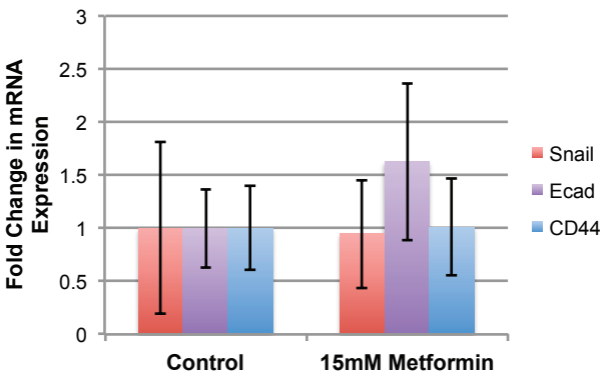

Supplement: Additional File 1 — Format: PDF. Cell death does not significantly alter expression of EMT and stem cell genes in JLO-1. A drug control was used to confirm that dose-dependent induction of EMT genes and repression of stem cell genes was not a mere epiphenomenon of cell death accompanying salinomycin treatment. We have previously discovered that Metformin does not influence EMT in JLO-1 cells at non-cytotoxic doses, indicating it does not regulate EMT in JLO-1 (unpublished data). (A) An MTS assay was initially performed to determine the cytoxicity curve for JLO-1 cells treated with Metformin for 72 hours. (B) At non–cytotoxic concentrations, Metformin does not regulate EMT based on RT-qPCR data of Snail and E-cadherin transcript levels, thus it is an appropriate drug control to induce cell death in JLO-1. (C) Upon 48-hour treatment of JLO-1 with 15 mM Metformin to induce approximately 60% cell death (equivalent to the cell death observed from 4 μM salinomycin treatment), expression of EMT genes Snail and E-cadherin showed only minor changes. In addition, CD44 expression was not effected by induction of cell death. [file 1471-2407-12-556-S1.pdf]
